# Ovarian Vein Thrombosis as a Complication of Laparoscopic Surgery

**DOI:** 10.1155/2015/682941

**Published:** 2015-12-14

**Authors:** Anu Gupta, Natasha Gupta, Josef Blankstein, Richard Trester

**Affiliations:** Department of Obstetrics and Gynecology, Mount Sinai Hospital, 1500 S California Avenue, Chicago, IL 60608, USA

## Abstract

Ovarian vein thrombosis (OVT) is an extremely rare but life-threatening complication of the postpartum period. It has never been reported as a complication of laparoscopic surgery. We report a case of right ovarian vein thrombosis that occurred in the postoperative period after patient underwent laparoscopic salpingectomy for a right side ectopic pregnancy. She presented with 1-week history of abdominal pain and fever. A complete workup for fever was performed and was found negative. Computed tomography of the abdomen and pelvis revealed right ovarian vein thrombosis. The patient was treated with anticoagulant therapy and responded well.

## 1. Introduction

The diagnosis of OVT is a hard nut to crack secondary to its rarity. OVT is usually diagnosed in the postpartum period but may also occur secondary to pelvic inflammatory disease, malignancy, and pelvic surgery. It is usually suspected when patient presents with fever or abdominal pain unresponsive to conservative treatment. We report here a patient who presented with persistent fever and abdominal pain of unknown cause, following a laparoscopic right salpingectomy. When all the workup for fever was noted negative, patient underwent a computed tomography (CT) scan of abdomen and pelvis that detected a thrombus in the right ovarian vein. CT scan of abdomen and pelvis is the diagnostic modality of choice where a thrombus is noted as a central hypodense area in an enlarged ovarian vein. OVT is rarely reported after laparoscopic procedures and in this era when minimally invasive surgeries are on hike, its diagnosis should be considered with higher suspicion. We also emphasize the importance of paying attention to ovarian veins during a CT examination, especially in the background of risk factors and suspicious symptomatology as a small clot can be easily missed.

## 2. Case Presentation

A 41-year-old woman Gravid 4 Para 0 presented to emergency room with complaints of lower abdominal pain and fever for the last 1 week. She also gave a recent history of ectopic pregnancy for which she underwent a laparoscopic right salpingectomy at another hospital. She denied any nausea, vomiting, vaginal bleeding, vaginal discharge, or diarrhea and declined any significant medical problems or medication use. Patient had a history of three spontaneous abortions and one ectopic pregnancy treated with laparoscopic right salpingectomy, 2 weeks back. She denied any other surgical history and also denied any workup for recurrent first trimester miscarriages. She was currently trying to conceive for the last two years. Patient is a nonsmoker and denied any personal or family history of bleeding or thrombotic disorders.

Upon examination, her abdomen was nontender, nondistended, and without any palpable masses. The rest of the physical examination including inspection of surgical wound, Homans' signs, and Moses' sign was negative. The pelvic examination revealed an anteverted, normal size, nontender uterus, without any adnexal tenderness or palpable masses. Patient was febrile at 101 degrees but other vital signs were within normal limits. Urine pregnancy test was negative. Laboratory examination showed white blood cell count of 13.4 × 10^9^/L with 78% neutrophils and elevated C-reactive protein of 120 mg/dL. The rest of the complete blood count and comprehensive metabolic profile were within normal limits.

**Figure 1:**
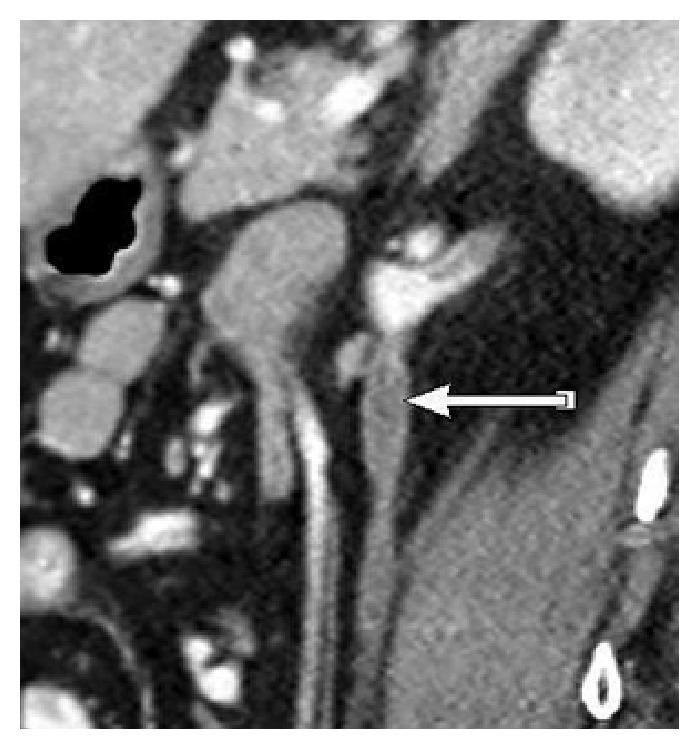
Coronal view of contrast enhanced CT in the portal venous phase, depicting enlarged right ovarian vein with central hypodense lesion, suggestive of ovarian vein thrombosis.

**Figure 2:**
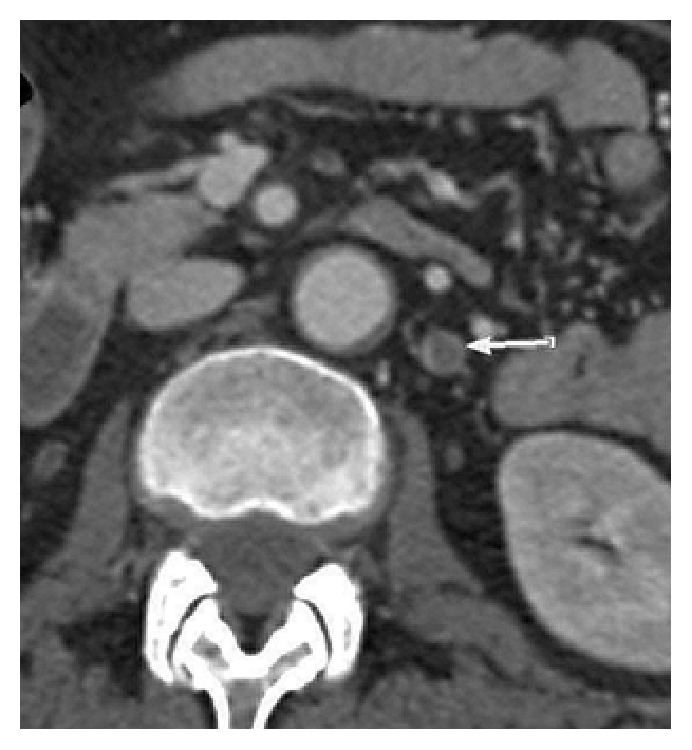
Axial view of CT showing wall enhancement of right ovarian vein in the portal venous phase, with a low density lesion representing the thrombus.

The remaining workup for fever, including chest X-ray and urinalysis, was noted negative. Patient was admitted with the diagnosis of “fever of unknown etiology” and started on antibiotics. Blood cultures were sent prior to initiating treatment with antibiotics. A hypercoagulable workup performed due to patient's history of recurrent spontaneous abortions returned negative. CT scan of the abdomen and pelvis with intravenous contrast was performed on hospital day 1, which revealed right ovarian vein thrombosis, without extension into the inferior vena cava (IVC) (Figures 1 and 2).

Treatment with therapeutic dose of low molecular weight heparin (Enoxaparin 60 mg, subcutaneous injection twice a day) was started. Patient received Enoxaparin for 2 weeks, following which oral anticoagulation with Warfarin was initiated. Warfarin was continued for 6 months with close outpatient follow-ups. Patient responded very well to the treatment and a follow-up CT revealed absence of thrombus in the right ovarian vein. Warfarin was discontinued at this time and patient resumed care with maternal fetal medicine for preconceptional counseling and management during next pregnancy.

## 3. Discussion

OVT is a rare venous thromboembolic disease of puerperium that affects 0.05–0.18% of postpartum women [1]. The underlying pathophysiology of OVT in pregnancy is attributed to Virchow's triad of venous stasis, endothelial damage, and hypercoagulability [2, 3]. Other conditions responsible for causing thrombus formation in ovarian vein are malignancies, pelvic inflammatory diseases, sepsis, and pelvic surgeries. It may rarely be idiopathic and occur spontaneously without any identifiable risk factors or underlying hypercoagulable disorders [4, 5]. Thrombus occurs in the right ovarian vein in 70–90% of cases, although some authors have reported higher incidence in left ovarian vein as well [2, 6]. This increased incidence on right side is due to the longer length and absence of competent valves in right ovarian vein [7]. Bilateral thrombi are seen in 11–14% of cases [7].

The diagnosis of ovarian vein thrombosis is difficult due to the nonspecific symptoms of abdominal pain, flank pain, fever, nausea, vomiting, and rarely an abdominal mass. It may mimic a myriad of conditions like acute appendicitis, pyelonephritis, ovarian torsion, pelvic inflammatory disease, tuboovarian abscess, or inflammatory bowel disease [7, 8]. Often, an incidental diagnosis of OVT is made when CT scan of abdomen and pelvis is performed to establish the cause of patient's symptoms. Our patient also presented with nonspecific symptoms of abdominal pain and fever, 2 weeks after a laparoscopic surgery. She was treated conservatively but continued to have persistent symptoms, leading to a CT scan of abdomen and pelvis.  Figure 1 is the coronal image and Figure 2 depicts the axial view of an enhancing wall and a low attenuation thrombus in the right ovarian vein (Figures 1 and 2).

Contrast enhanced CT of abdomen and pelvis is considered the diagnostic modality of choice when OVT is suspected [9]. Other imaging studies available to examine the ovarian veins include magnetic resonance angiography (MRA) and color Doppler ultrasound. MRA offers several advantages over CT in diagnosis of OVT. It avoids ionizing radiation, has sensitivity and specificity approaching 100% compared to CT with sensitivity of 77.8% and specificity of 62.5%, and avoids intravenous contrast administration due to the use of diffusion weighted imaging [7, 9, 10]. Despite these benefits, many physicians use CT as the first imaging tool due to its easy availability. USG with Doppler may be used for follow-up of OVT patients after the treatment is initiated; its sensitivity in diagnosis of OVT is 52% and is by far poorer than CT or MRA [2, 11].

Our patient also gave a history of three first trimester spontaneous abortions; thus a full laboratory investigation for thrombophilias was performed, including factor V Leiden gene mutation, prothrombin gene mutation, protein-C and protein-S activity, MTHFR gene mutation, hyperhomocysteinemia, systemic lupus erythematosus, and antiphospholipid syndrome. Although the workup resulted negative, we believe that her history of recurrent abortions may have some deportment on current diagnosis of OVT as both the conditions are frequently a result of underlying hypercoagulable disorder. Additionally, this patient presented 2 weeks after a laparoscopic right salpingectomy which was likely the inciting factor for OVT and hitherto not recorded in the literature.

OVT is treated most commonly with anticoagulation therapy. There are no standard guidelines for the dose, duration, or agent of choice for anticoagulation. Most authors treat their patient for 3–6 months, where therapeutic dose of unfractionated heparin or LMWH (Enoxaparin or Dalteparin) is used for acute treatment and vitamin K antagonist (Warfarin) for maintenance of oral anticoagulation with INR maintained between 2 and 3 [1, 2, 12, 13]. Rivaroxaban (a heparinoid oral anticoagulant; direct factor Xa inhibitor) has also been proposed as an alternative to vitamin K antagonists for the long-term treatment of OVT [14]. Antibiotics are added where septic pelvic thrombophlebitis is suspected, as apparent from patient's persistent spike in fevers [3, 15]. Antibiotics are used for duration ranging from 48 hours up to 7 days, depending on the response to treatment. Various antibiotics proposed for septic pelvic thrombophlebitis include a beta-lactam antibiotic with beta-lactamase inhibitor, a second-third generation cephalosporin with metronidazole, clindamycin, or gentamicin [11].

Like any other venous thromboembolic disease, OVT can prove fatal and lead to serious complications like pulmonary embolism, sepsis, extension of thrombus into IVC, or renal vein [1, 16]. These can be managed using catheter directed thrombolysis with tPA, angioplasty of the renal vein, and thrombectomy or with an IVC filter [11, 14]. We believe that timely diagnosis and management of OVT can help prevent these potentially life-threatening complications. A high index of suspicion is needed when diagnosing OVT, especially following a laparoscopic procedure due to its rarity and casual symptomatology. This requires that special attention be paid to ovarian veins during CT imaging of suitable patients. This case is an attempt to bring to attention the possibility of OVT outside of puerperium, as a potential postoperative complication of minimally invasive surgeries.

## 4. Conclusion

OVT is a rare complication of pelvic surgeries, especially minimally invasive procedures. Its diagnosis is difficult due to lack of specific symptomatology and low incidence. CT with contrast enhancement can facilitate the diagnosis when ovarian veins are closely examined. Given the increasing trend of laparoscopic procedures, we emphasize that diagnosis of OVT should be considered in patients, presenting after a laparoscopic procedure and unresponsive to routine conservative treatment.
